# Infestation patterns of *Ixodes scapularis* and *Dermacentor variabilis* on dogs and cats across Canada

**DOI:** 10.1371/journal.pone.0281192

**Published:** 2023-02-02

**Authors:** Sydney DeWinter, Cathy Bauman, Andrew Peregine, J. Scott Weese, Katie M. Clow

**Affiliations:** 1 Department of Population Medicine, Ontario Veterinary College, University of Guelph, Guelph, Ontario, Canada; 2 Department of Pathobiology, Ontario Veterinary College, University of Guelph, Guelph, Ontario, Canada; University of Kentucky College of Medicine, UNITED STATES

## Abstract

Due to recent climatic and land use changes, Canada has experienced changes in tick populations, leading to an increased risk of tick bites and tick-borne pathogen exposure, especially in eastern Canada. Preventative recommendations for companion animals from veterinary professionals include regular use of tick prevention products and tick checks. Tick checks, specifically, should target regions of an animal’s body which are deemed to be high risk for tick attachment. However, tick species-specific infestation patterns on dogs and cats are largely understudied, and additional research is needed to help guide targeted tick checks. The objective of this study was to identify tick species-specific infestation patterns on dogs and cats. Ticks were collected for one year (April 2019 –March 2020) from 94 veterinary clinics across Canada as part of the Canadian Pet Tick Survey. All ticks were identified to species, and data on the location of tick attachment were ascertained with each submission. To examine the association between location of attachment (outcome) and tick species (explanatory variable), specifically *Ixodes scapularis* and *Dermacentor variabilis*, mixed effects univariable models were built. Two thousand three hundred and six submissions were received from 1925 dogs and 381 cats across Canada. Of these submissions, 1377 comprised *Ixodes scapularis*, and 620 comprised *Dermacentor variabilis*. Clear tick species-specific infestation patterns for dogs were present, with *I*. *scapularis* being significantly more likely to be found on the shoulders, and *D*. *variabilis* more likely to be found on the ears and neck. *Dermacentor variabilis* was more likely to be found on the cranial aspect of cats’ limbs, compared to *I*. *scapularis*. Up-to-date information on infestation patterns can be used to inform veterinary professionals and pet owners of common attachment sites based on established ticks in their region and thus conduct targeted tick checks.

## Introduction

Canada is currently experiencing changes in the abundance, distribution, and seasonality of several tick species. These shifts may increase the risk of tick bites, leading to subsequent pathogen exposure and disease development in companion animals [1]. Some tick species, such as *Ixodes scapularis* and *Dermacentor variabilis*, are vectors of several disease-causing pathogens in humans and animals [2–4]. In Canada, serum antibodies to *Borrelia burgdorferi*, a bacterial spirochete that is transmitted by *I*. *scapularis* and causes Lyme disease, have been consistently identified in dogs, particularly in central and eastern provinces [1, 4–6]. Evaluation of canine serological testing data over an 8-year period (2008–2015) showed significant increases in pathogen prevalence over time in the provinces of Manitoba, Ontario, Quebec, and Nova Scotia [1]. Clearly, risk for tick-borne pathogen exposure and subsequent disease development is increasing across central and eastern Canada [1].

Due to the risks associated with tick bites, measures are recommended to prevent tick acquisition. Avoidance of tick habitat is the first recommendation, although this may be difficult depending on the companion animal’s lifestyle. Tick control products are also widely recommended [7–9] and either repel ticks or kill them while feeding [9]. Tick checks, which involve the owner running their fingers through a pet’s fur in search of ticks, can help find ticks before they attach or soon after. The time required for pathogen transmission following a tick bite is highly variable, but for many pathogens, the tick must feed for at least 24 hours. *Ixodes scapularis*, for example, requires at least 24 hours to transmit *B*. *burgdorferi* and *D*. *variabilis* must remain attached to the host for at least 24 hours to transmit *Rickettsia rickettsii* [4, 10]. Given this time window, owners are encouraged to check their pets daily, or after any outdoor activity, and remove ticks when they are found [9].

Preliminary evidence suggests that some tick species have preferential attachment sites on the body of companion animals. Little and colleagues [11] found an association with *I*. *scapularis* and bites on the head and neck, and *D*. *variabilis* on the head, neck and back of dogs and cats. Knowledge of tick attachment patterns and the tick species present in a given area allows pet owners to target relevant parts of their pet’s body within 24 hours of attachment and thus increase the likelihood of removing an infectious tick before a pathogen can be transmitted [9].

The Canadian Pet Tick Survey (CPTS) was a passive surveillance study that collected ticks from dogs and cats through participating veterinary clinics across Canada from April 2019 –March 2020. Using the resultant data, the objective of this study was to describe tick species-specific infestation patterns on Canadian cats and dogs, specifically those of *I*. *scapularis* and *D*. *variabilis*.

## Materials and methods

### Data collection

In February 2019, veterinary clinics across all Canadian provinces were invited to participate in this study (i.e., Canadian Pet Tick Survey). Details regarding the study were distributed via veterinary associations, educational websites (e.g., www.petsandticks.com), and public speaking engagements (e.g., Ontario Veterinary Medical Association). Clinics were enrolled on a first come first-serve basis in each province until baseline targets were reached (i.e., each province had a specific target number of clinics with which to enroll based on geographic size and population).

Participating clinics submitted all ticks collected on companion animals for one year (April 1, 2019 to March 31, 2020). A questionnaire, which included information on the animal as well as the location of the tick bite(s), accompanied each submission (S1 Appendix). For this study, a submission was defined as all the ticks collected from a single animal at a single point in time. As tick samples used in this study were secondary use, no animal use protocol was required. The questionnaire only collected information related to the animal and thus research ethic board approval for research involving human participants was not required. There was no cost for clinics to participate in this study.

### Tick identification

Following removal from the dog or cat host and prior to shipment, ticks were stored in the refrigerator with damp cotton balls to prevent desiccation of ticks. Upon arrival to the laboratory, ticks were placed in 70% ethanol and identified to species using a stereomicroscope and standard keys [4, 12]. *Dermacentor* spp. identification for specimens from British Columbia, Alberta, and Saskatchewan were confirmed by PCR. This additional step was taken as morphological appearance is similar between *D*. *variabilis* and *D*. *andersoni* populations are known to overlap in these areas [13, 14]. DNA was extracted using the DNeasy Blood and Tissue Kit^TM^ (Qiagen) as per manufacturer’s protocol. The second internal transcribed spacer ribosomal DNA (ITS-2) was amplified, as described by Dergousoff and Chilton [13]. Individual amplicons were differentiated based on size using gel electrophoresis, and species was determined based on band size [13]. Ticks which were unable to be identified to species using a stereomicroscope and standard keys (or, if they were *Dermacentor* spp., through PCR), were subsequently removed from analysis.

### Data cleaning

Data were compiled in Microsoft Excel version 16.57 (https://office.microsoft.com/excel; 2021) and cleaned using OpenRefine version 3.4.1 (https://openrefine.org/#; 2020). Any submission that contained multiple tick species (co-infestation) was excluded because it was not possible to elucidate which tick species was attached to each site based on the questionnaire data.

**Fig 1:**
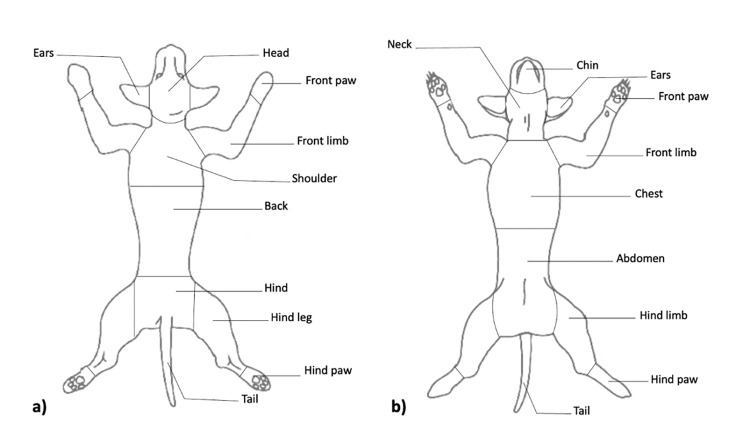
Diagram provided to illustrate the location of the tick bite on the animal’s body. Veterinarians or pet owners indicated the dorsal (a) and/or ventral (b) location of the tick bite by marking an ‘x’ on the diagram. Image obtained from Midwestern University Animal Health Institute (https://www.mwuanimalhealth.com; 2022).

The initial locations of the tick bites on the body were compiled based on biological relevance (Fig 1).

Dorsally, responses ‘ears’, and ‘head’ were combined to form the category ‘head’, and ‘hind’ and ‘tail’ were combined to form the category ‘hind’. Cranially, ‘front limb’, ‘front paw’, ‘hind leg/limb’, and ‘hind paw’ were combined to form the category ‘limbs cranial’. ‘Back’ and ‘shoulder’ were not combined further during analysis. Ventrally, responses ‘abdomen’ and ‘chest’ were combined to form the category ‘underbelly’, ‘chin’ and ‘neck’ were combined to form the category ‘neck’; and caudally ‘front limb’, ‘front paw’, ‘hind limb’ and ‘hind paw’ were combined to form the category ‘limbs caudal’. The response ‘ears’ was not combined further, and ‘tail’ was excluded from analysis due to a low number of submissions being received, and inability to combine with a biologically relevant category. Submissions which recorded more than one tick of the same species were recorded as ‘multiple dorsal’ or ‘multiple ventral’ if location of ticks on the body varied (Table 1).

**Table 1:** Categorization of locations of tick attachment.

Categorized location of attachment		Original location of attachment (as per Fig 1)
Dorsal	Head	Ears, head
	Hind	Hind, tail
	Shoulder	Shoulder
	Back	Back
	Multiple	Multiple
Cranial	Limbs	Front limb, front paw, hind leg/limb, hind paw
Ventral	Underbelly	Abdomen, chest
	Neck	Chin, neck
	Ears	Ears
	Multiple	Multiple
Caudal	Limbs	Front limb, front paw, hind limb, hind paw

The location of the tick bite was recorded on a diagram for each dog and cat included in the study. Following data cleaning, location of attachment was categorized to dorsal and ventral areas based on biological relevance.

### Statistical analyses

Data were separated by dog and cat. Descriptive statistics (e.g., counts and ranges), were produced to generate summary tables and visualizations of the data. Univariable mixed effect logistic regression models, with a random effect for veterinary clinic, were conducted to examine the association between the location on the animal’s body (outcome) and the tick species (*I*. *scapularis* or *D*. *variabilis)*. The outcome variable was categorized as yes (location of interest) and no (all other locations). Tick species, the explanatory variable, was categorized as *I*. *scapularis* (yes) or no (*D*. *variabilis*). The referent category was changed when a significant association was found with *D*. *variabilis* to express odd ratios >1. As dog size varies greatly based on breed, breed group was further explored through 2x2 contingency tables and interaction terms. A significance level of *α*≤0.05 guided the interpretation of the associations. All statistical analyses were conducted in STATA version 17.0 (STATACorp, College Station, TX, USA; 2021).

## Results

### Tick collection

**Fig 2:**
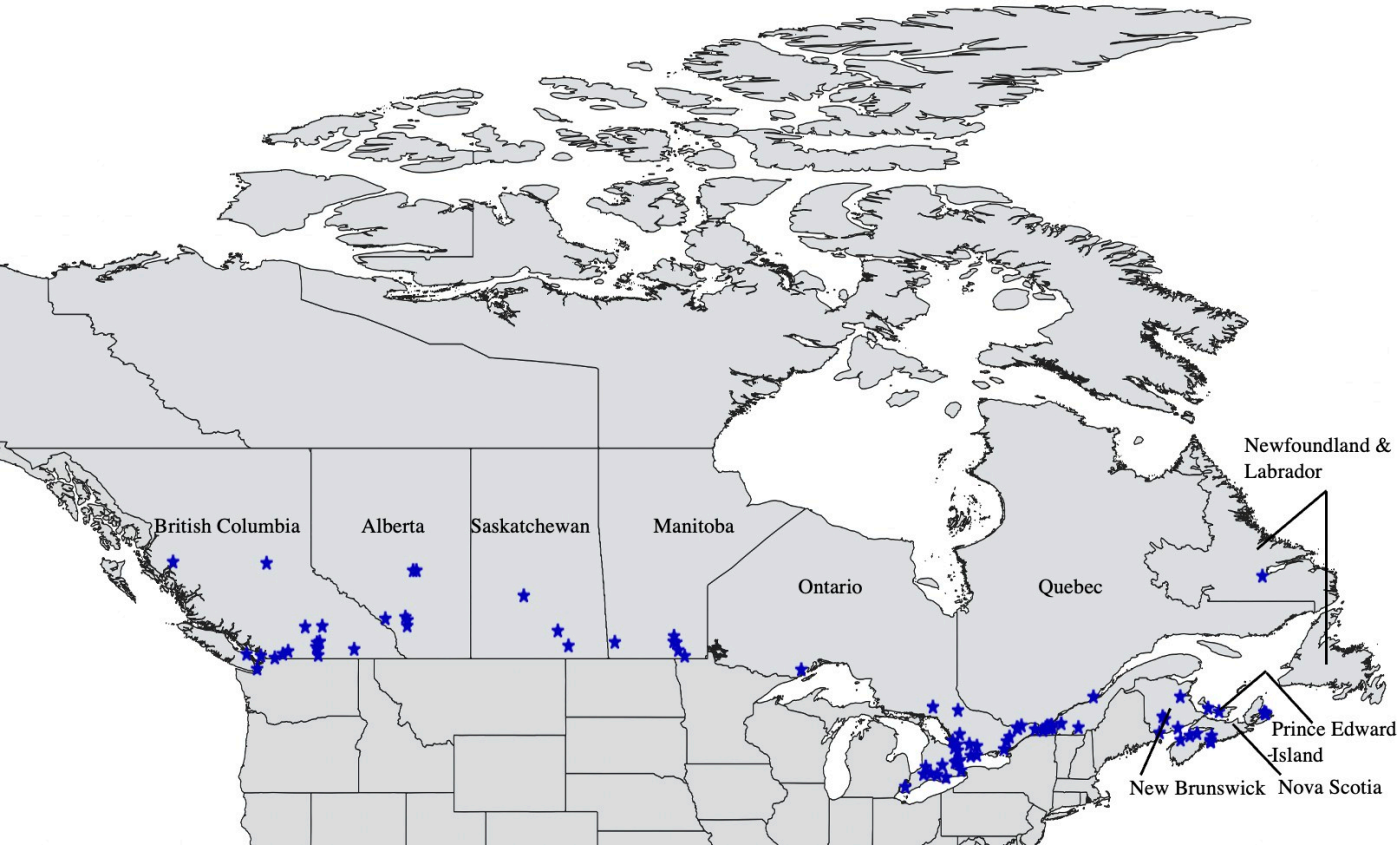
Geographic locations of veterinary clinics participating in the Canadian Pet Tick Survey (blue stars) across Canadian provinces. (Spatial data were prepared using QGIS version 3.22.1 ((https://qgis.org/en/site/; 2022). Base vector layers were ascertained through the Scholars GeoPortal at the University of Guelph (http://geo1.scholarsportal.info).

Ninety-four clinics enrolled in the study, and all provinces in Canada were represented (Fig 2). Two thousand three hundred and six submissions were included in analyses, with tick species from five genera being represented: *Ixodes* spp. (n = 1597 submissions), *Dermacentor* spp. (n = 689), *Rhipicephalus* sp. (n = 16), *Amblyomma* spp. (n = 3) and *Haemaphysalis* sp. (n = 1). The vast majority of tick submissions were *I*. *scapularis* (59.71%, n = 1377) or *D*. *variabilis* (26.88%, n = 620). Submissions with coinfestations (n = 50) and ticks which could not be identified to species (n = 19) were not included in analyses. The tick species and life stages collected, the spatial and temporal patterns of submissions, and the demographic data on the population of companion animals included in the study have been reported elsewhere [15].

Ticks were submitted from 1925 dog submissions (83%) and 381 cat submissions (17%). Of these submissions, 1997 were contained either *I*. *scapularis* or *D*. *variabilis*, with 1686 submissions from dogs and 311 from cats. Most of these submissions (76.8%) were single tick submissions, and 91.6% of submissions contained ≤3 ticks. Tick bite location was provided for 1895 submissions, with 1590 submissions from dogs and 305 submissions from cats. Contingency 2x2 tables were generated to investigate breed group, but there was not sufficient data to include breed group in the model (i.e., many cells contained <10 submissions).

### Canine infestation patterns

**Fig 3:**
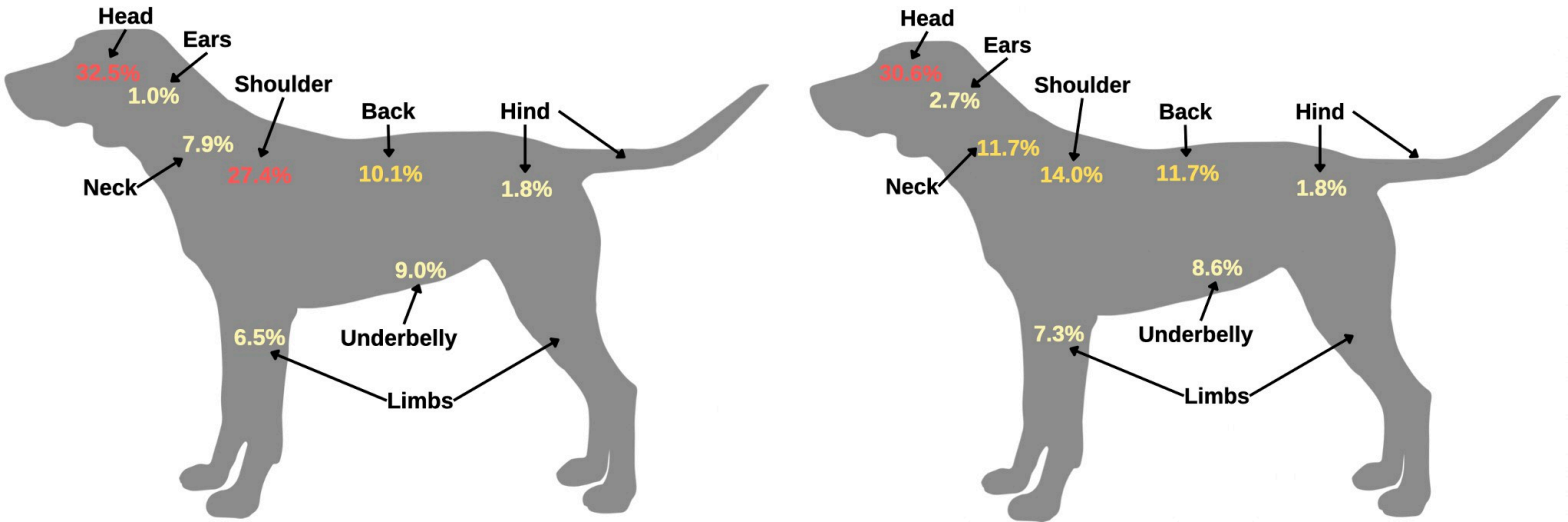
Distribution of attachment sites of ticks on dogs. A. *Ixodes scapularis* B. *Dermacentor variabilis*. Diagrams were generated in Canva (https://www.canva.com; 2022). Note: percentages will not total 100% because some submissions had multiple tick bite locations.

**Fig 4:**
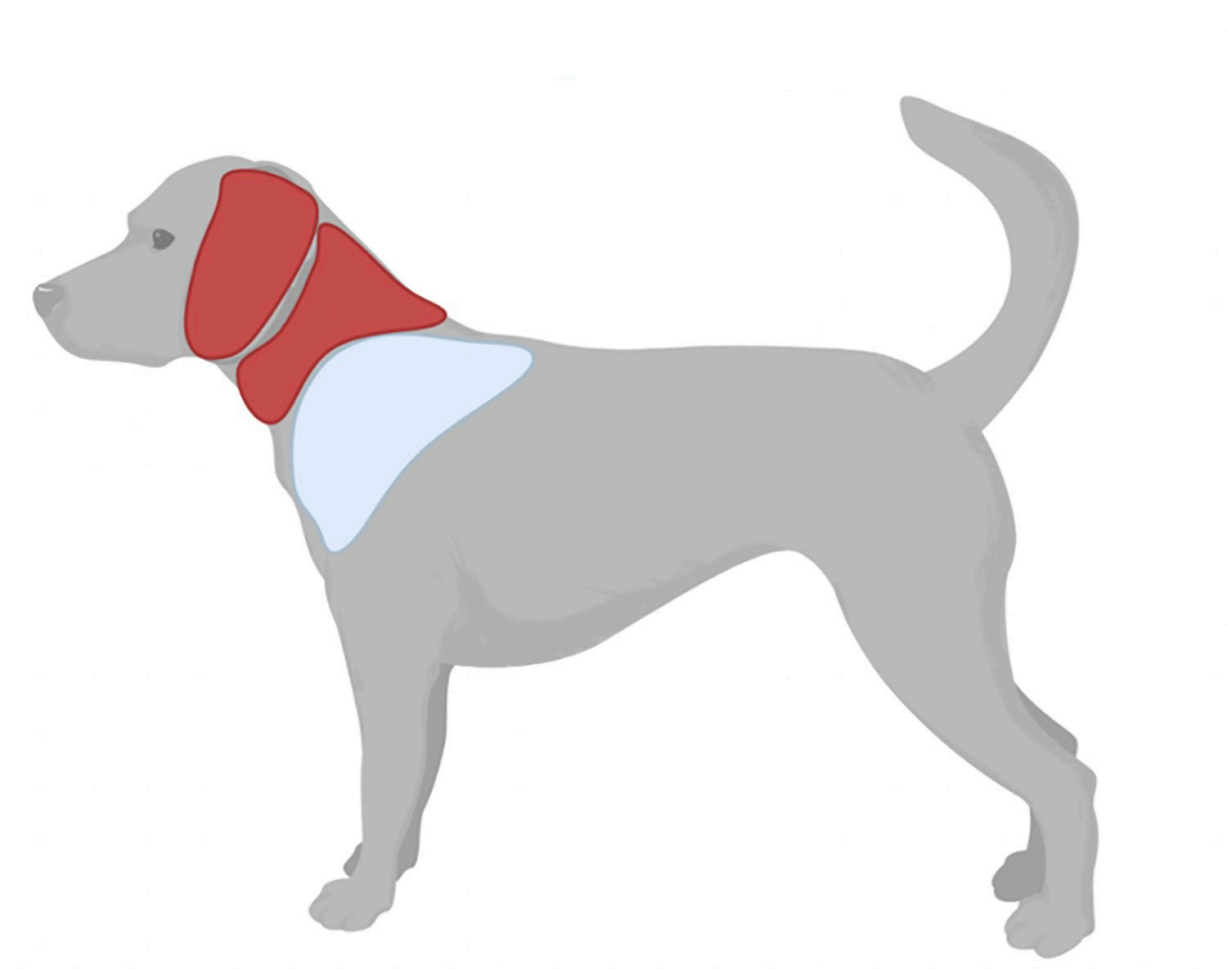
Odds of tick attachment on sites of the dog body. Red represents the locations of the body with a significantly higher odds of *Dermacentor variabilis* acquisition, and where the blue represents the locations of the body with a significantly higher odds of *Ixodes scapularis* acquisition. Diagrams were generated in BioRender (https://biorender.com; 2022).

*Ixodes scapularis* (n = 1044 dog submissions) was found attached both dorsally (n = 835 submissions) and ventrally (n = 209 submissions). Dorsally, *I*. *scapularis* was most frequently attached to the head (n = 339; 32.5%), shoulder (n = 286; 27.4%), and back (n = 105; 10.1%) of the dog. Ventrally, *I*. *scapularis* was found attached frequently to the underbelly (n = 94; 9.0%) and the neck (n = 83; 7.9%) of the dog (Table 2). The odds of finding a tick on the shoulder was 2.11 times (95% CI = 1.53–2.91; *p* < 0.001) higher for *I*. *scapularis*, compared to *D*. *variabilis* (Figs 3 and 4; Table 3).

**Table 2:** Descriptive statistics of tick bite location on dogs and cats.

Tick Species	Location of attachment		Dog	Cat
			No. Submissions	No. Submissions
*Ixodes scapularis*	Dorsal	Head	339	53
		Hind	19	0
		Shoulder	286	124
		Back	105	14
		Multiple	47	12
	Cranial	Limbs	39	2
	Ventral	Underbelly	94	7
		Neck	83	55
		Ears	10	5
		Multiple	9	1
	Caudal	Limbs	12	1
*Dermacentor variabilis*	Dorsal	Head	167	7
		Hind	10	0
		Shoulder	76	10
		Back	64	2
		Multiple	48	2
	Cranial	Limbs	29	2
	Ventral	Underbelly	47	3
		Neck	64	4
		Ears	15	0
		Tail	1	0
		Multiple	15	1
	Caudal	Limbs	11	0

**Table 3:** Mixed univariable logistic regression analysis of tick bite location on dogs. This regression explored the association between tick bite location (outcome) and tick species (*Ixodes scapularis* vs. *Dermacentor variabilis*) on dogs across Canada.

		Odds Ratio (OR)	95% Confidence Interval (CI)	*p*-value
**Dorsal—Head**				
Tick Species	*I*. *scapularis*	1.12	0.90–1.41	0.295
**Cranial–Limbs**				
Tick Species	*I*. *scapularis*	0.73	0.43–1.25	0.259
**Dorsal–Shoulder**				
Tick Species	*I*. *scapularis*	2.11	1.53–2.91	<0.001
**Dorsal—Back**				
Tick Species	*I*. *scapularis*	0.87	0.62–1.23	0.441
**Dorsal–Multiple**				
Tick Species	*D*. *variabilis*	1.91	1.17–3.11	0.009
**Ventral–Hind**				
Tick Species	*I*. *scapularis*	1.01	0.46–2.27	0.967
**Ventral–Underbelly**				
Tick Species	*I*. *scapularis*	1.07	0.75–1.55	0.688
**Ventral–Ears**				
Tick Species	*D*. *variabilis*	3.13	1.30–7.55	0.011
**Caudal–Limbs**				
Tick Species	*I*. *scapularis*	0.62	0.21–1.86	0.396
**Ventral–Neck**				
Tick Species	*D*. *variabilis*	1.49	1.06–2.10	0.022
**Ventral–Multiple**				
Tick Species	*D*. *variabilis*	3.16	1.38–7.28	0.007

*Dermacentor variabilis* (n = 546 dog submissions) was also found attached both dorsally (n = 394 dog submissions) and ventrally (n = 152 dog submissions) on dogs. Dorsally, *D*. *variabilis* was most frequently attached to the head (n = 167; 30.6%), shoulder (n = 76; 13.9%), and the back (n = 64; 11.7%) of the dog. Ventrally, *D*. *variabilis* was found attached frequently to the underbelly (n = 47; 8.6%) and the neck (n = 64; 11.7%) of the dog (Table 3). The odds of tick attachment on the ears (OR = 3.13; 95% CI = 1.30–7.55; *p* = 0.011) and neck (OR = 1.49; 95% CI = 1.06–2.10; *p* = 0.022) of the dog were significantly higher than other locations of the animal’s body, for *D*. *variabilis*, compared to *I*. *scapularis*. Infestation in multiple locations of the body, both dorsally (OR = 1.91; 95% CI = 1.17–3.11; *p* = 0.009) and ventrally (OR = 3.16; 95% CI = 1.38–7.28; *p* = 0.007), were significantly higher for *D*. *variabilis* (Figs 3 and 4; Table 3).

### Feline infestation patterns

*Ixodes scapularis* (n = 274 cat submissions) was found attached both dorsally (n = 205), and ventrally (n = 69). Dorsally, *I*. *scapularis* was found most frequently on the shoulder (n = 124; 45.3%) of the cat. Ventrally, *I*. *scapularis* was found attached frequently to the neck (n = 55; 20.1%) of the cat (Table 2).

**Fig 5:**
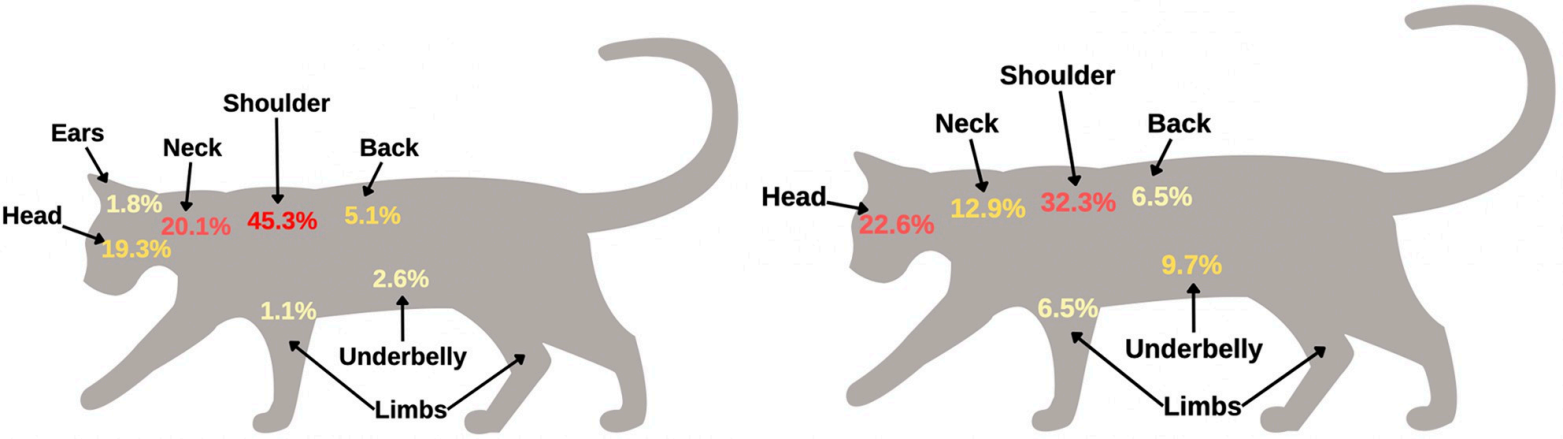
Distribution of attachment sites of ticks on cats. A. *Ixodes scapularis* B. *Dermacentor variabilis*. Diagrams were generated in Canva (https://www.canva.com; 2022). Note: percentages will not total 100% because some submissions had multiple tick bite locations.

**Fig 6:**
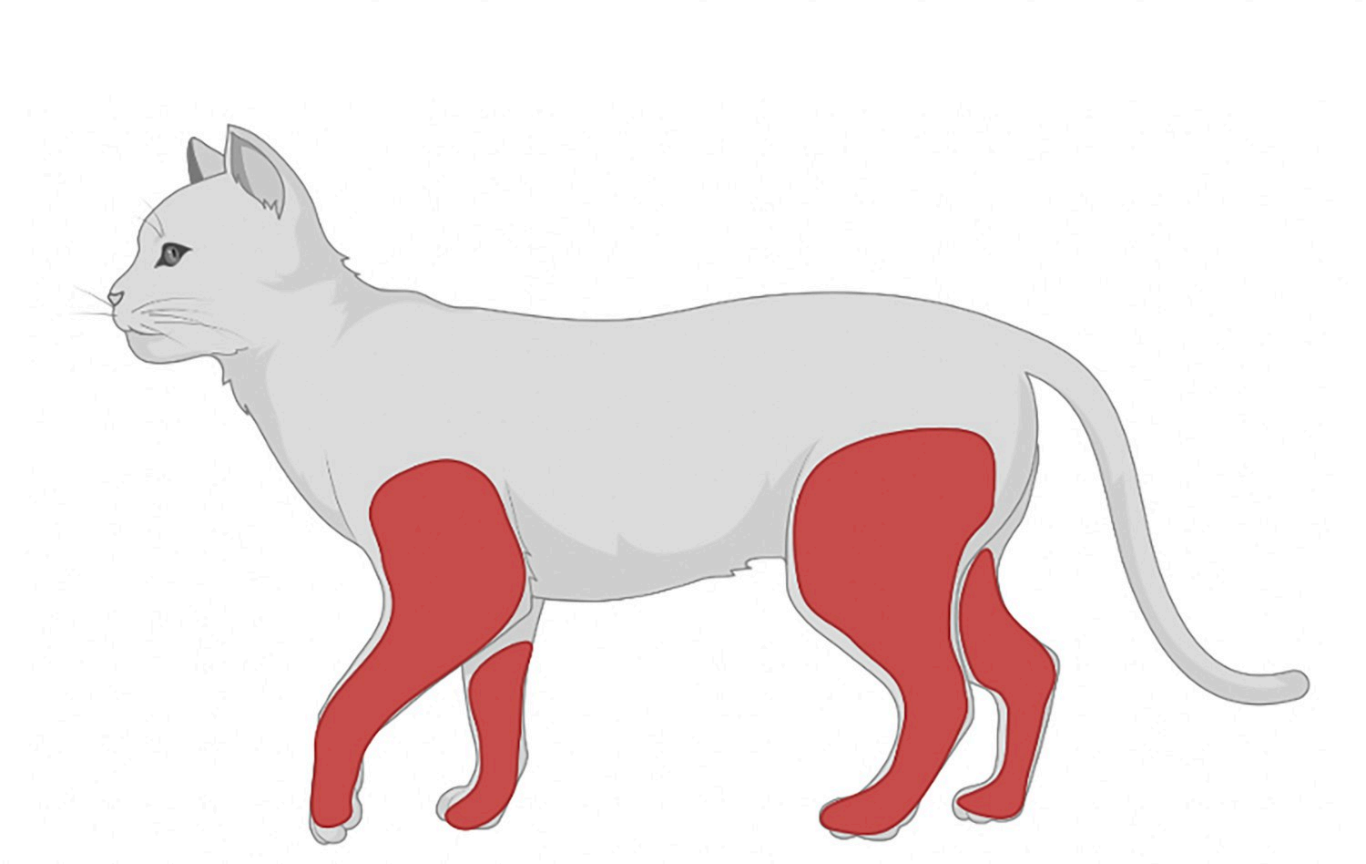
Odds of tick attachment on sites of the cat body. Red represents the locations of the body with a significantly higher odds of *Dermacentor variabilis* acquisition. Diagrams were generated in BioRender (https://biorender.com; 2022).

There were only 31 submissions available for *D*. *variabilis* on cats; 23 submissions were dorsal and 8 submissions were ventral. Dorsally, *D*. *variabilis* was most attached to the shoulder (n = 10; 32.3%), of the cat. Ventrally, there were few *D*. *variabilis* submissions, and no distinct patterns could be elucidated (Table 2). The odds of finding a tick on the cranial aspect of the cat’s limbs (OR = 9.23; 95% CI = 1.25–67.92; *p* = 0.029), were significantly higher for *D*. *variabilis*, compared to *I*. *scapularis* (Figs 5 and 6; Table 4).

**Table 4:** Mixed univariable logistic regression analysis of tick bite location on cats. This regression explored the association between bite location (outcome) and tick species (*Ixodes scapularis* vs. *Dermacentor variabilis*) on cats across Canada.

		Odds Ratio (OR)	95% Confidence Interval (CI)	*p*-value
**Dorsal—Head**				
Tick Species	*I*. *scapularis*	0.83	0.33–2.07	0.690
**Cranial–Limbs**				
Tick Species	*D*. *variabilis*	9.23	1.25–67.93	0.029
**Dorsal–Shoulder**				
Tick Species	*I*. *scapularis*	1.72	0.75–3.95	0.200
**Dorsal—Back**				
Tick Species	*I*. *scapularis*	0.79	0.17–3.66	0.766
**Dorsal–Multiple**				
Tick Species	*I*. *scapularis*	0.65	0.13–3.42	0.615
**Ventral–Underbelly**				
Tick Species	*D*. *variabilis*	4.92	0.90–27.03	0.066
**Ventral–Neck**				
Tick Species	*I*. *scapularis*	1.77	0.58–5.45	0.318
**Ventral–Multiple**				
Tick Species	*I*. *scapularis*	0.11	0.01–1.92	0.131

## Discussion

The risk of tick acquisition, and subsequent development of tick-borne disease has been established as a concern for Canadian companion animals [1, 5]. Preventative measures recommended by veterinarians, which include conducting tick checks, have been shown to be effective in preventing tick acquisition and the development of tick-borne disease [7–9]. In this study, the infestation patterns were explored for two tick species of companion animal health significance, *I*. *scapularis* and *D*. *variabilis*, on dogs and cats, to help enhance the targeted application of tick checks.

For dogs, *I*. *scapularis* and *D*. *variabilis* tick bites were commonly reported on the head, shoulders, and back. *Ixodes scapularis* was more likely to be found on the shoulders of dogs when compared to *D*. *variabilis*, while *D*. *variabilis* was more likely to be found on the ears and neck of dogs, compared to *I*. *scapularis*. Infestation of *D*. *variabilis* on more than one location of the dog was more likely, compared *I*. *scapularis*. For cats, the shoulders were also a common location for both *I*. *scapularis* and *D*. *variabilis* tick bites. *Dermacentor variabilis* was more likely to be found on the cranial aspects of cats’ limbs, compared to *I*. *scapularis*.

Previous studies have identified the head and neck of the animal as common sites of *I*. *scapularis* and *D*. *variabilis* attachment, similar to the descriptive analyses of this study [11, 16]. Further, previous research also found that the ears of dogs were more likely to be infested with *D*. *variabilis*. Interestingly, this study found the limbs of cats to be more likely to be infested with *D*. *variabilis* than *I*. *scapularis*, while research by Little and colleagues [11], found this site to have a very low attachment preference for both species of tick (i.e., *I*. *scapularis* and *D*. *variabilis*). It should be noted, however, that there are limited studies on this topic, and further elucidation of these patterns will only be possible if future studies are conducted.

Infestation patterns may partially be explained by considering places on an animal where ticks can easily access while questing [17]. For example, while dogs and cats are sniffing in the grasses or bushes, they may acquire ticks around their head, neck, and ears [4]. In addition, regions around the collar of the animal (e.g., the shoulders) can harbour ticks, which is likely because dogs and cats are unlikely to reach this area when grooming [11]. These explanations only apply to general infestation patterns, though, not species-specific patterns, and it is still unknown if there are biological reasons associated with each tick species that influence tick bite location.

Integration of previous spatial and temporal analyses of tick populations, along with the results described here, can be used to inform pet owners and veterinarians of regions on an animal’s body to inspect for ticks, based on geographic location and time of year. Spatially, in Canada, *I*. *scapularis* and *D*. *variabilis* can be found in central and eastern provinces [2–4, 15, 18–20]. Adult *Ixodes scapularis* are most active in the spring (April–May) and fall months (October–November), and adult *D*. *variabilis* are most active in the spring and early summer [2–4, 15, 18–20].

Tick checks on companion animals are recommended widely [7, 21, 22]. Specifically, the Canadian Parasitology Expert Panel recommends client education and tick checks, followed by the use of a tick prevention product [21]. Currently, there is limited research surrounding the percentage of veterinarians that recommend tick checks in Canada. However, previous studies have indicated that most veterinarians practicing in Canada discuss ticks and tick-borne disease with their clients routinely and do recommend tick prevention [23]. There is also limited research regarding tick check uptake in owners (i.e., how many owners conduct tick checks, and how often), even if this is recommended by veterinarians. Uptake and compliance to these measures and recommendations should be explored in future studies.

While this study has provided valuable insight into tick infestation patterns, there are limitations that should be acknowledged. Selection (volunteer) bias is likely, as clinics were enrolled on a volunteer basis and may have been motivated to do so based on regional tick presence and risk. For example, there was a high level of clinic enrollment in eastern provinces, specifically Ontario, where *I*. *scapularis* is established and of concern. Data for this study were obtained from an owner-reported questionnaire that was completed at the time of tick submission. However, many owners did not complete the questionnaire in detail, limiting the level of detail available for data analysis. Moreover, some ticks were dead upon collection from the prevention products that the animals were receiving, and some ticks were only crawling on the animal or recently attached (unengorged) and found via a tick check. Therefore, location of tick attachment may have been reported as where the tick was found crawling and thus not represent a location of attachment. Further, due to the nature of tick collection and submission, submissions from dogs and cats, which were co-infested with multiple species of tick were removed from analysis, thus reducing the power to detect differences. While dog size varies greatly depending on the breed, this study did not have sufficient data to further explore how this may impact the location of tick attachment.

This study identified infestation patterns of *I*. *scapularis* and *D*. *variabilis*. When veterinary practitioners explain appropriate tick checks for these tick species, they can recommend that owners pay particular attention to the head, ears, and shoulders. In the future, additional research is needed to build on these findings to further assess the infestation patterns of *I*. *scapularis* and *D*. *variabilis* on cats and infestation patterns of other tick species of veterinary significance. Additional research is also recommended to explore the impact that dog size has on infestation patterns.

## Supporting information

S1 Appendix (DOCX)

## References

[1] Evason M, Stull J. W, Pearl D. L, Peregrine A. S, Jardine C, Buch J. S, et al. (2019). Prevalence of *Borrelia burgdorferi*, *Anaplasma* spp., *Ehrlichia* spp. and *Dirofilaria immitis* in Canadian dogs, 2008 to 2015: a repeat cross-sectional study. Parasit Vectors. 2019;12(64). 10.1186/s13071-019-3299-9 PMC6350403 30691522

[2] Dantas-Torres F, Chomel B. B, Otranto D. Ticks and tick-borne diseases: A One Health perspective. Trends Parasitol. 2012;28: 437–446. doi: 10.1016/j.pt.2012.07.003 22902521

[3] Estrada-Peña A, de la Fuente J. The ecology of ticks and epidemiology of tick-borne viral diseases. Antiviral Res. 2014;108: 104–128. doi: 10.1016/j.antiviral.2014.05.016 24925264

[4] Lindquist E. E, Wu K. W, Flahey B. A Handbook to the Ticks of Canada (Ixodida: Ixodidae, Argasidae). Biological Survey of Canada; 2016. pp. 36–54; 64–83; 127–167.

[5] Herrin B. H, Peregrine A. S, Goring J, Beall M. J, Little S. E. Canine infection with *Borrelia burgdorferi*, *Dirofilaria immitis*, *Anaplasma* spp. and *Ehrlichia* spp. in Canada, 2013–2014. Parasit Vectors. 2017;10(244). 10.1186/s13071-017-2184-7 PMC5437676 28526093

[6] Jones T. F, Garman R. L, LaFleur B, Stephan S. J, Schaffner W. Risk factors for tick exposure and suboptimal adherence to preventive recommendations. Am J Prev Med. 2002;23: 47–50. doi: 10.1016/s0749-3797(02)00440-3 12093423

[7] Littman M. P, Gerber B, Goldstein R. E, Labato M. A, Lappin M. R, Moore G. E. ACVIM consensus update on Lyme borreliosis in dogs and cats. J Vet Intern Med. 2018;32: 887–903. doi: 10.1111/jvim.15085 29566442 PMC5980284

[8] Littman M. P, Gerber B, Goldstein R. E, Labato M. A, Lappin M. R, Moore G. E. ACVIM Small animal consensus statement on Lyme disease in dogs: Diagnosis, treatment, and prevention. J Vet Intern Med. 2006;20: 422–434. doi: 10.1892/0891-6640(2006)20[422:asacso]2.0.co;2 16594606

[9] Wright I, Cull B, Gillingham E. L, Hansford K. M, Medlock J. Be tick aware: when and where to check cats and dogs for ticks. Vet Rec. 2018;182: 514–517. doi: 10.1136/vr.104649 29483148

[10] Richards S, Langley R, Apperson C, Watson E. Do tick attachment times vary between different tick-pathogen systems? Environments 2017;4(37). 10.3390/environments4020037

[11] Little S. E, Barrett A. W, Nagamori Y, Herrin B. H, Normile D, Heaney K, et al. Ticks from cats in the United States: Patterns of infestation and infection with pathogens. Vet Parasitol. 2018;257: 15–20. doi: 10.1016/j.vetpar.2018.05.002 29907187

[12] Keirans J. E, Litwak T. R. Pictorial key to the adults of hard ticks, family Ixodidae (Ixodida: Ixodoidea), east of the Mississippi River. J Med Entomol. 1989;26: 435–448. doi: 10.1093/jmedent/26.5.435 2795615

[13] Dergousoff S. J, Chilton N. B. Differentiation of three species of ixodid tick, *Dermacentor andersoni*, *D*. *variabilis* and *D*. *albipictus*, by PCR-based approaches using markers in ribosomal DNA. Mol Cell Probes. 2007;21: 343–348. 10.1016/j.mcp.2007.04.003 17544620

[14] Dergousoff S. J, Galloway T. D, Lindsay L. R, Curry P. S, Chilton N. B. Range expansion of *Dermacentor variabilis* and *Dermacentor andersoni* (Acari: Ixodidae) near their northern distributional limits. J Med Entomol. 2013;50: 510–520. 10.1603/me12193 23802445

[15] DeWinter S, Bauman C, Peregrine A, Weese J. S, Clow K. M. Assessing the spatial and temporal patterns and risk factors for acquisition of Ixodes spp. by companion animals across Canada. Ticks Tick Borne Dis. 2023;14: 102089. 10.1016/j.ttbdis.2022.102089 36423538

[16] Saleh M. N, Sundstrom K. D, Duncan K. T, Ientile M. M, Jordy J, Ghosh P, et al. Show us your ticks: A survey of ticks infesting dogs and cats across the USA. Parasit Vectors. 2019;12(595). doi: 10.1186/s13071-019-3847-3 31856893 PMC6923977

[17] Meyers H. Seven places to look for ticks on your dog [Internet]. American Kennel Club. American Kennel Club; 2021 [cited 2022 September 25]. Available from: https://www.akc.org/expert-advice/health/places-to-look-for-ticks-on-dog/

[18] Alkishe A., Peterson A. T. Potential geographic distribution of *Ixodes cookei*, the vector of Powassan virus. J. Vector Ecol. 2021;46, 155–162. 10.52707/1081-1710-46.2.155 35230020

[19] Koch H. G. (1982). Seasonal incidence and attachment sites of ticks (Acari: Ixodidae) on domestic dogs in southeastern Oklahoma and northwestern Arkansas, USA. J Med Entomol. 1982;19: 293–298. doi: 10.1093/jmedent/19.3.293 7120309

[20] Lorusso V, Dantas-Torres F, Lia R. P, Tarallo V. D, Mencke N, Capelli G, et al. Seasonal dynamics of the brown dog tick, *Rhipicephalus sanguineus*, on a confined dog population in Italy. Med Vet Entomol. 2010;24: 309–315 10.1111/j.1365-2915.2010.00885.x 20557458

[21] Canadian Parasitology Expert Panel Guidelines [Internet]. Canadian Parasitology Expert Panel Guidelines—Canadian Parasitology Expert Panel Guidelines | CPEP—University of Saskatchewan. [cited 2022 September 25]. Available from: https://research-groups.usask.ca/cpep/index.php

[22] Preventing ticks on your pets [Internet]. Centers for Disease Control and Prevention. Centers for Disease Control and Prevention; 2019 [cited 2022 September 25]. Available from: https://www.cdc.gov/ticks/avoid/on_pets.html

[23] Nichol G. K, Weese J. S, Evason M, Clow K. M. Assessing knowledge, attitudes, and practices of Canadian veterinarians with regard to Lyme disease in dogs. J Vet Intern Med. 2021;35: 294–302. doi: 10.1111/jvim.16022 33421198 PMC7848372

