# Supplementary figures and images for: Infestation patterns of Ixodes scapularis and Dermacentor variabilis on dogs and cats across Canada
Source: PLoS One. 2023 Feb 2;18(2):e0281192. doi: 10.1371/journal.pone.0281192 (PMC9894407; doi:10.1371/journal.pone.0281192)

**Appendix A**


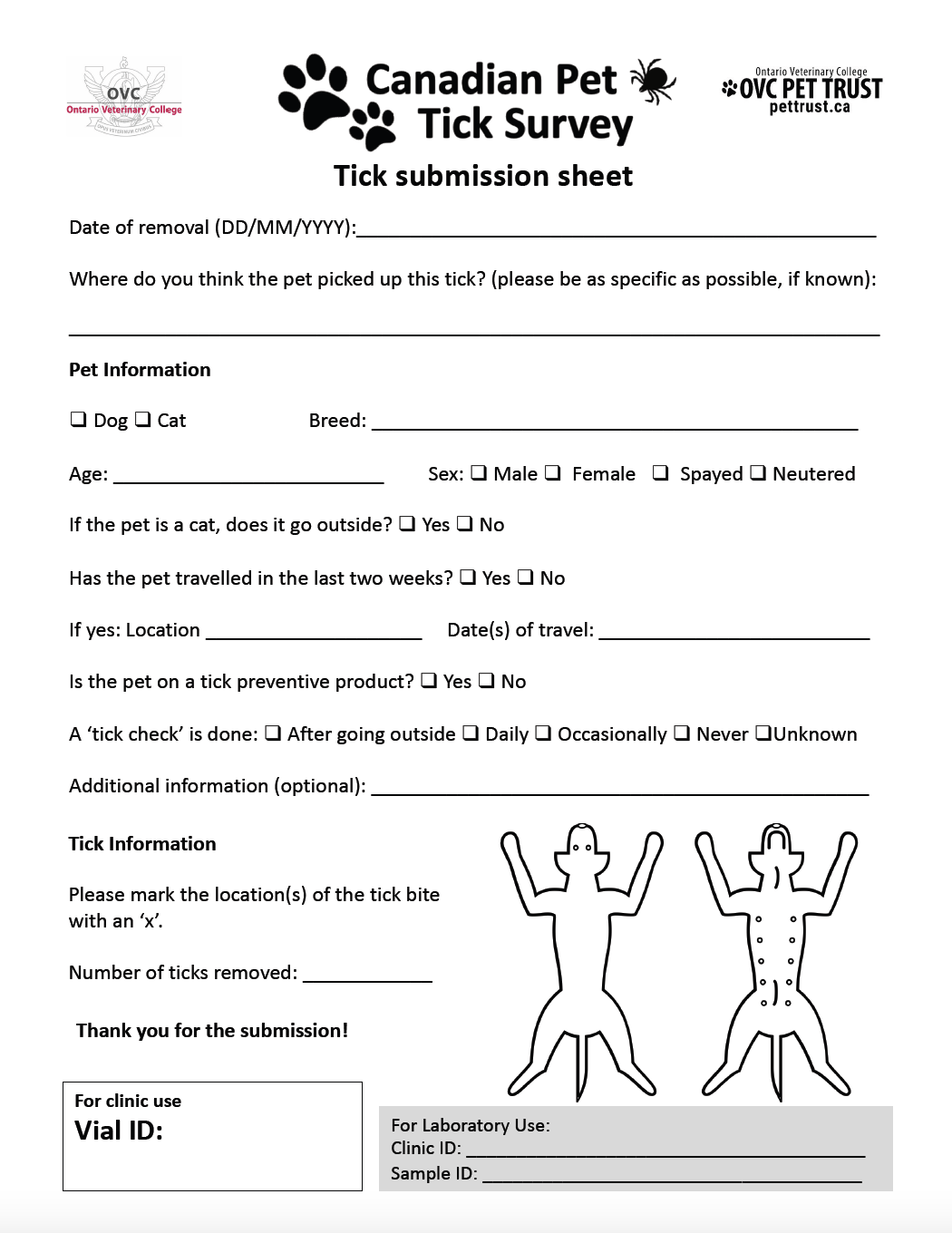

Supplement: S1 Appendix — (DOCX) [file pone.0281192.s001.docx]
